# Supplementary figures and images for: The elevated expression of ORF75, a KSHV lytic gene, in Kaposi sarcoma lesions is driven by a GC-rich DNA cis element in its promoter region
Source: PLoS Pathog. 2025 Mar 17;21(3):e1012984. doi: 10.1371/journal.ppat.1012984 (PMC11981178; doi:10.1371/journal.ppat.1012984)

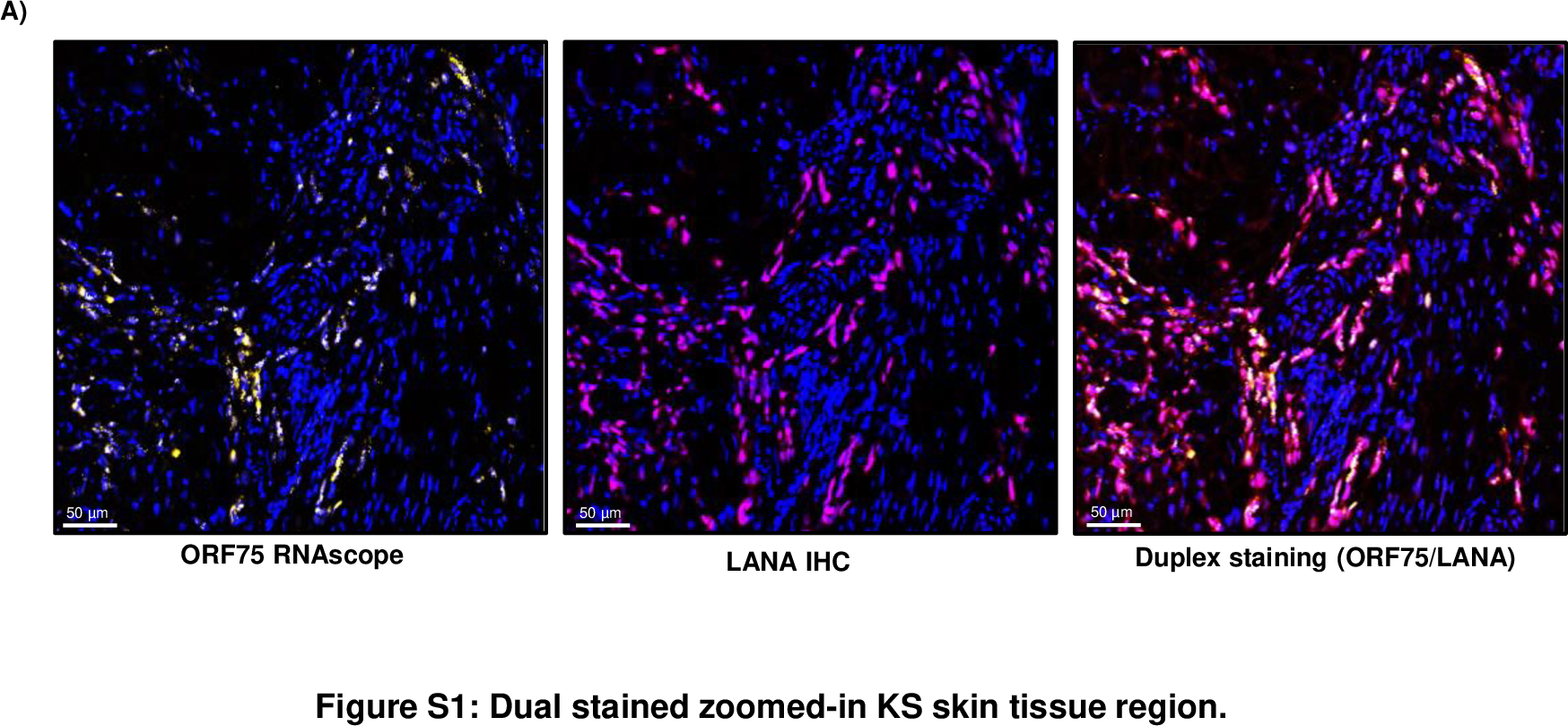

Supplement: S1 Fig — A) KS Skin lesion zoomed in section from panel A, showing colocalization of ORF75 RNA and LANA protein. (TIF) [file ppat.1012984.s001.tif]

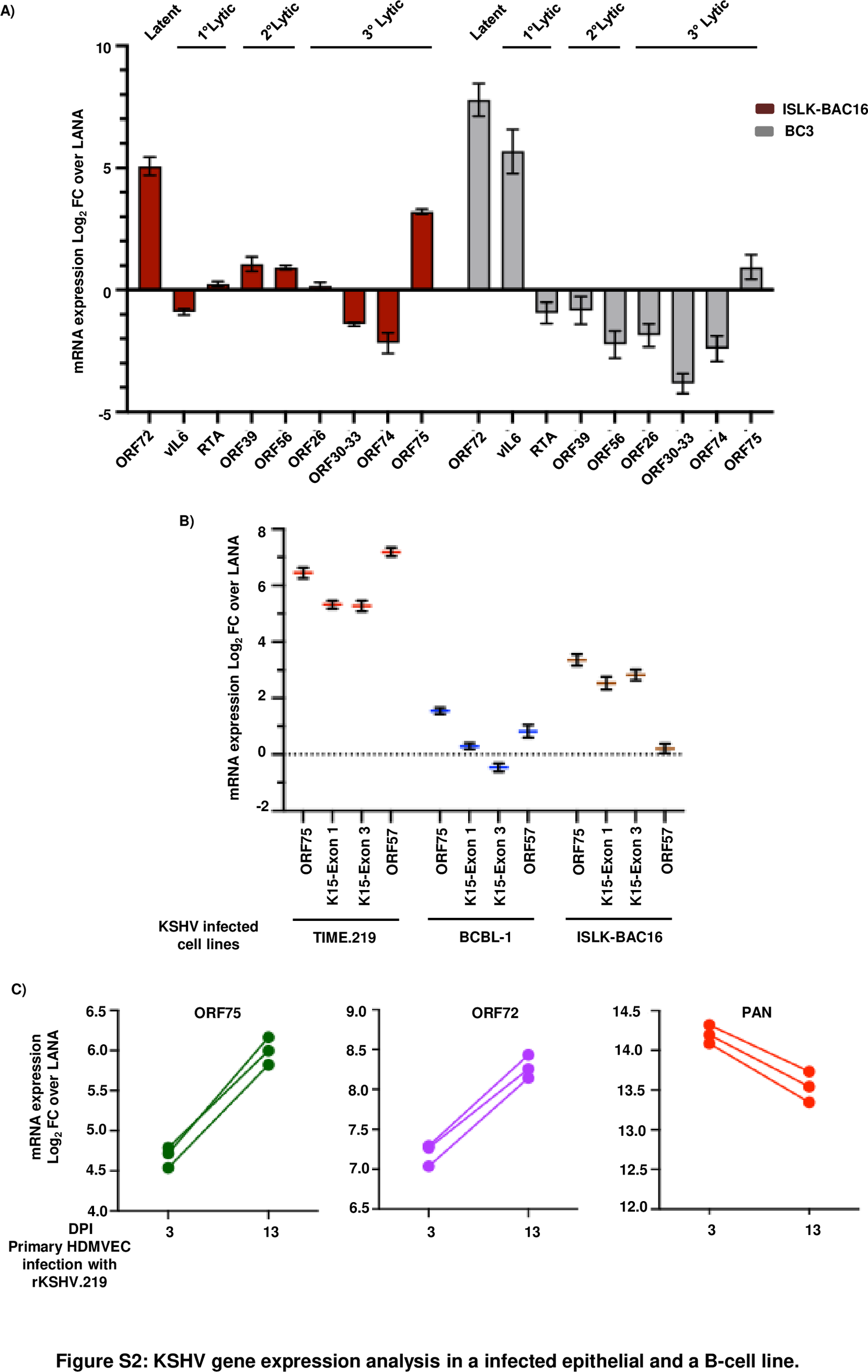

Supplement: S2 Fig — A) qPCR analysis of representative genes of latent and lytic cycle in latently infected iSLK-BAC16 and PEL cell line, BC3. N=3 biological replicate with three qPCR technical replicate. Expression of all genes were normalized to respective LANA expression for each cell type. Internal reference gene GAPDH. B) qPCR analysis of ORF75 RNA, K15 RNA using two distinct primer set targeting exon 1 and 3 of the K15 gene and ORF57 RNA in latently infected TIME.219, BCBL-1 and ISLK-BAC16 cells. C) qPCR analysis of de novo infection of primary Human Dermal Microvascular Endothelial cells (HDMVEC) with rKSHV.219 virus at MOI 1. Samples were collected at 3 and 13 days post infection. ORF72 and PAN RNA serves as a marker for latent and lytic infection cycle, respectively. Shown are the means ± standard deviations of at least 3 separate experiments. (TIF) [file ppat.1012984.s002.tif]

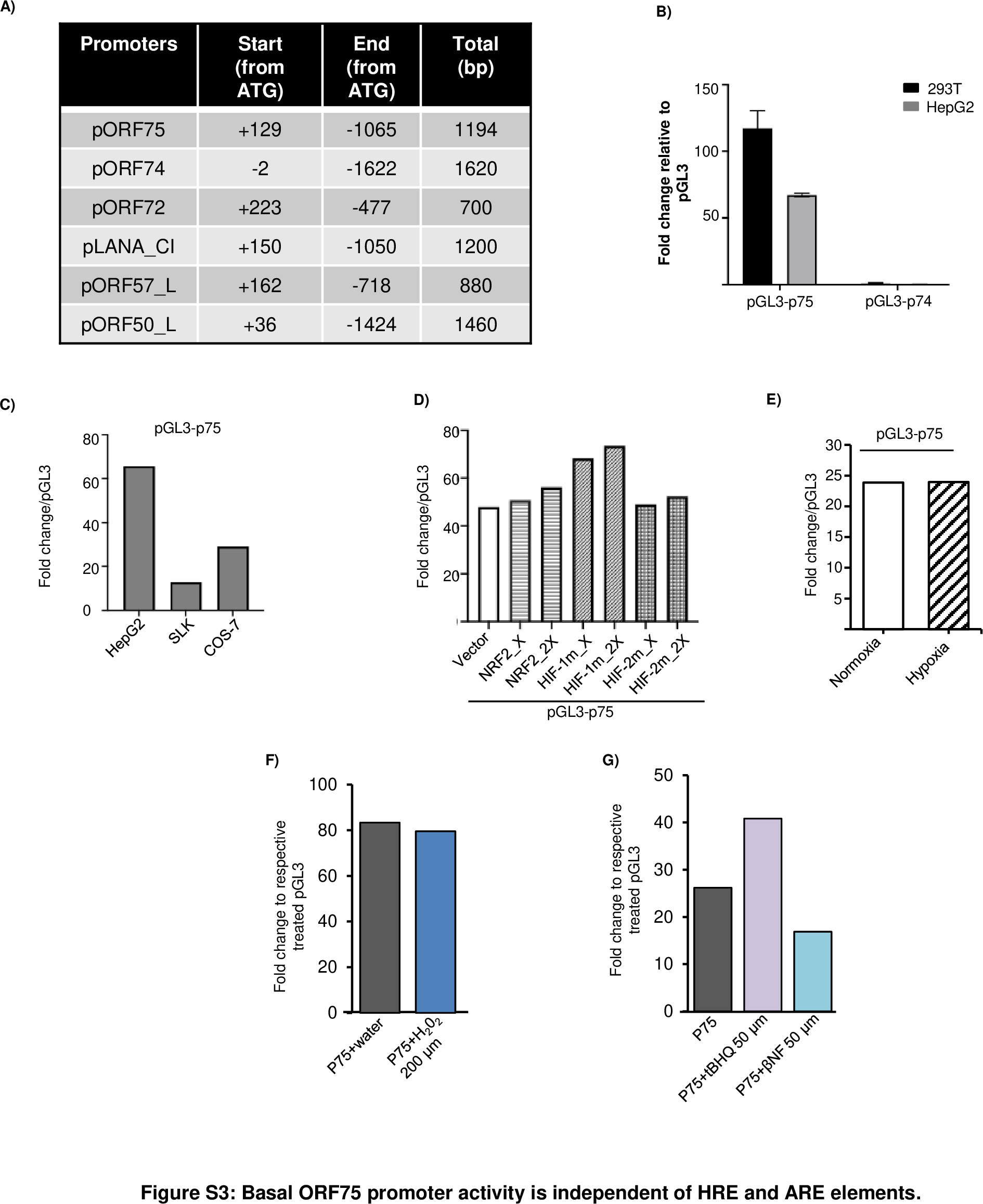

Supplement: S3 Fig — A) Table showing positions and lengths of different KSHV gene promoters used in Figs 3D and S3B. B) Promoter luciferase assay of ORF75 and ORF74 (1.2 kb) promoter in HEK293T and HepG2. Data normalised to respective pGL3 vector in each cell line. Histogram represents mean with SD as error bars for three biological replicate. Assayed at 72h post transfection. C) Promoter luciferase assay of ORF75 promoter in HepG2, SLK and COS-7 cells. Assayed at 72h post transfection. ORF75 promoter activity was normalized to pGL3 vector control activity set as 1 for each cell line. D) Promoter luciferase assay of the ORF75 promoter in HepG2 cells, coupled with transient overexpression of NRF2, HIF1α, and HIF-2α degradation-resistant mutants. X and 2X indicate 1:2 and 1:4 ratios of ORF75 promoter to protein expression plasmid, respectively. E, F, G) Promoter luciferase assay of ORF75 promoter in HepG2 cells with various treatments. All treatments were done 24h post transfection. Assayed at 48h. Histogram for all except B) is one experiment. (TIF) [file ppat.1012984.s003.tif]

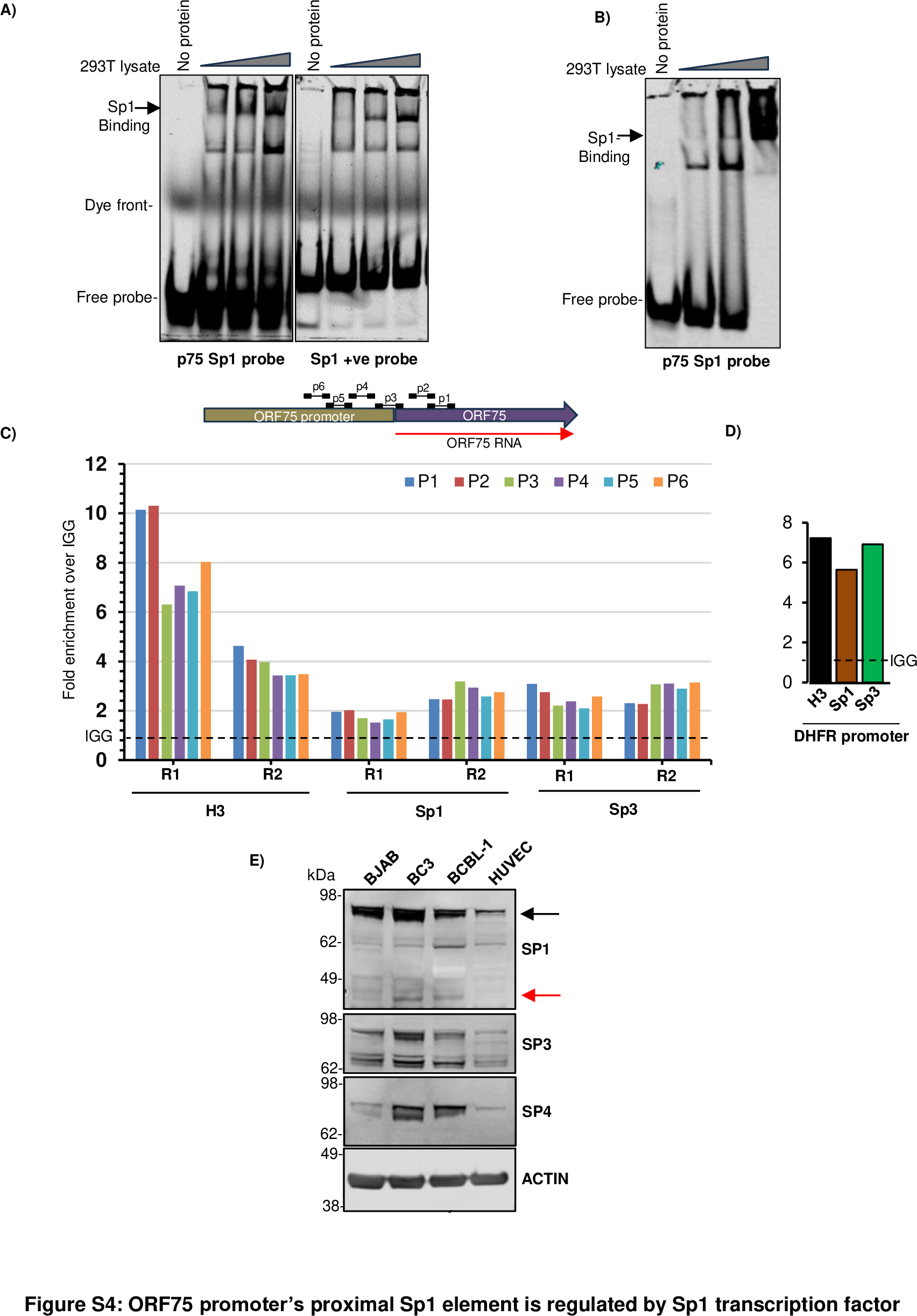

Supplement: S4 Fig — A) EMSA showing binding of Sp. proteins with dsDNA probe of ORF75 promoter along with a positive control Sp1 probe (Li-COR, P/N: 829-07926) in a 8% native PAGE gel. Three different protein concentrations used 10, 20 and 30 µg. B) Same as in A), except only p75-Sp1 probe used with three different concentrations of 293T whole cell lysates (10, 25 and 50 µg). C) ChIP assay analysing Sp1 and Sp3 binding regions of ORF75 promoters in latent iSLK-BAC16 cells. Binding was analysed by chromatin immunoprecipitation followed by qPCR. The schematic on the top shows the location of the various primers used to detect Sp protein abundance on the ORF75 promoter. H3 was used as a positive ChIP control and IGG antibody as isotype control. R1 and R2 are two separate experiments. D) Same as in C) except a 68 bp long DHFR gene promoter region located at -400 bp from transcription start site of the human DHFR gene was used as a positive control for Sp protein binding E) Western blot analysis of Sp1, Sp3 and Sp4 protein levels from different cell lines using whole cell lysates. Black and red arrow indicates full-length Sp1 and alternate SP1 forms in W.B, respectively. See S7 Fig for full blots. (TIF) [file ppat.1012984.s004.tif]

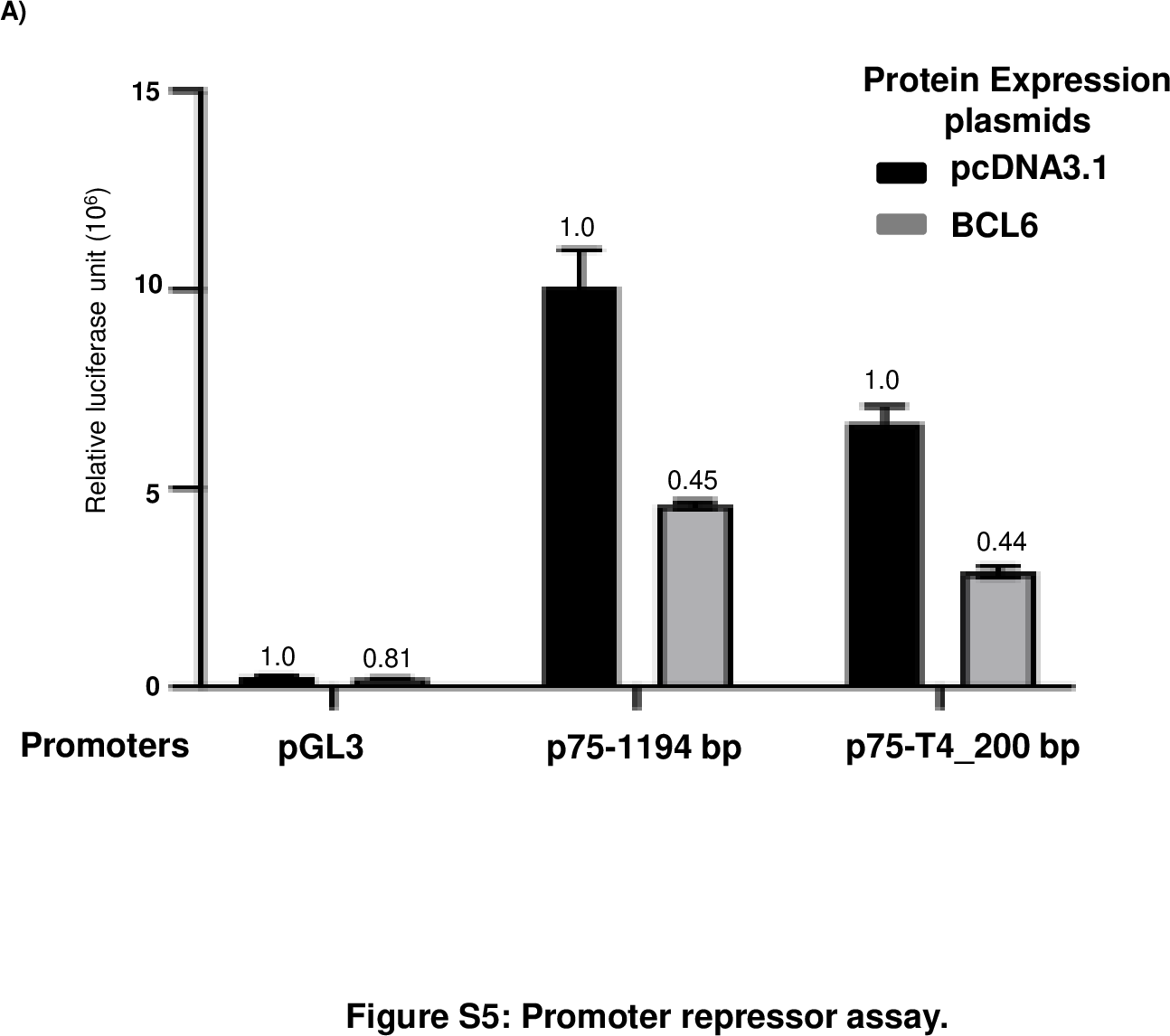

Supplement: S5 Fig — A) Promoter luciferase assay of full length ORF75 (p75) and truncated promoter construct (p75-T4) with co-expression of BCL6 expression constructs in 293T cell line. pcDNA3.1 plasmid was used as vector control for BCL6 expression plasmid. Assayed at 3 days post transfection. Error bar indicate ± standard deviations of 3 experiments. (TIF) [file ppat.1012984.s005.tif]

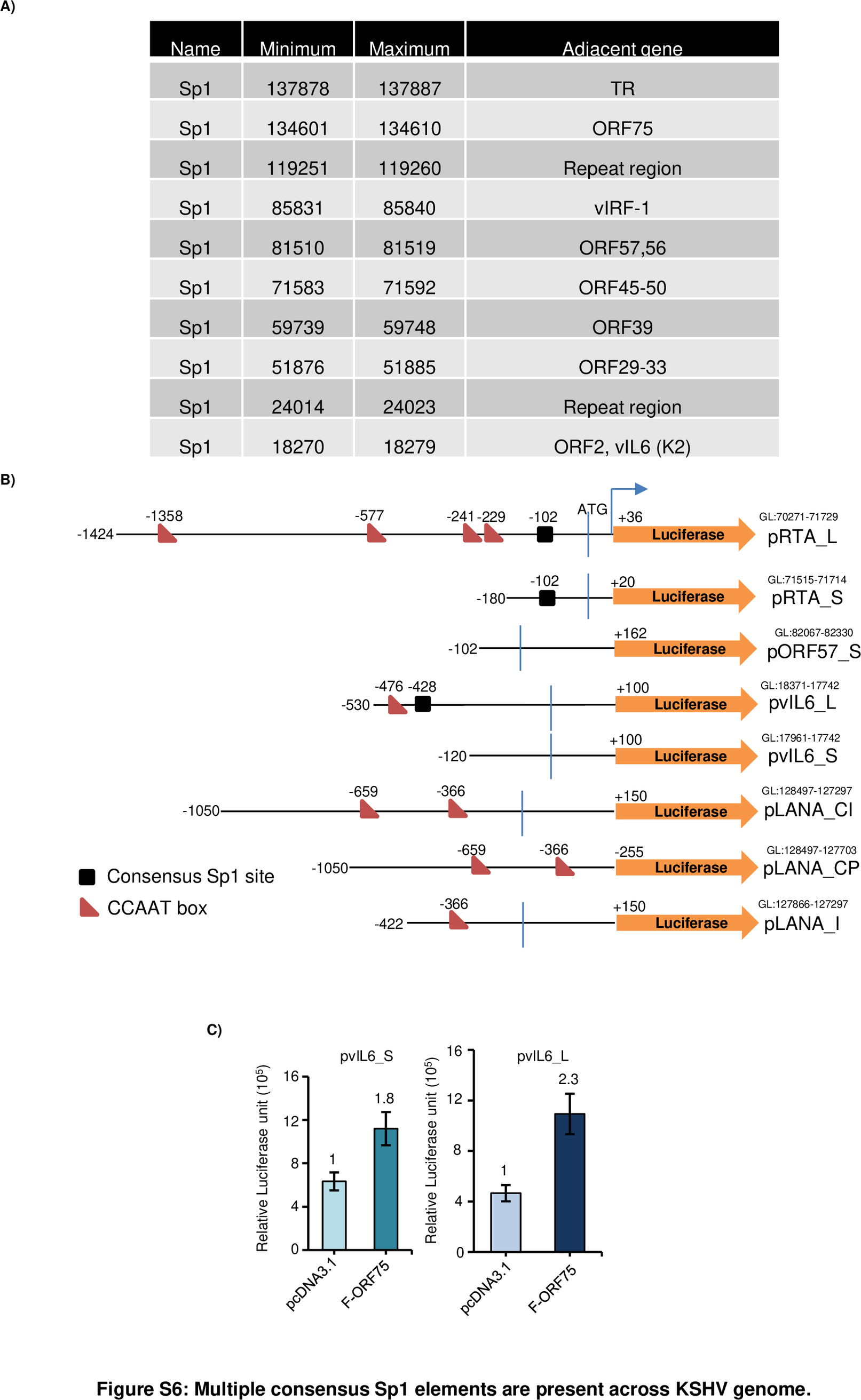

Supplement: S6 Fig — A) Table showing multiple consensus Sp1 element position throughout KSHV genome. Only complete consensus sequence is shown here. Consensus sequence KGGGCGGRRY, where K stands for G or T and R stands for G or A. B) Schematic diagram of various KSHV gene promoters used in this study. C) Promoter luciferase assay of vIL6 promoters along with co-expression of F-ORF75 protein in HEK293T cells. Assayed at 72h post transfection. Shown are the means ± standard deviations of 3 separate experiments. (TIF) [file ppat.1012984.s006.tif]

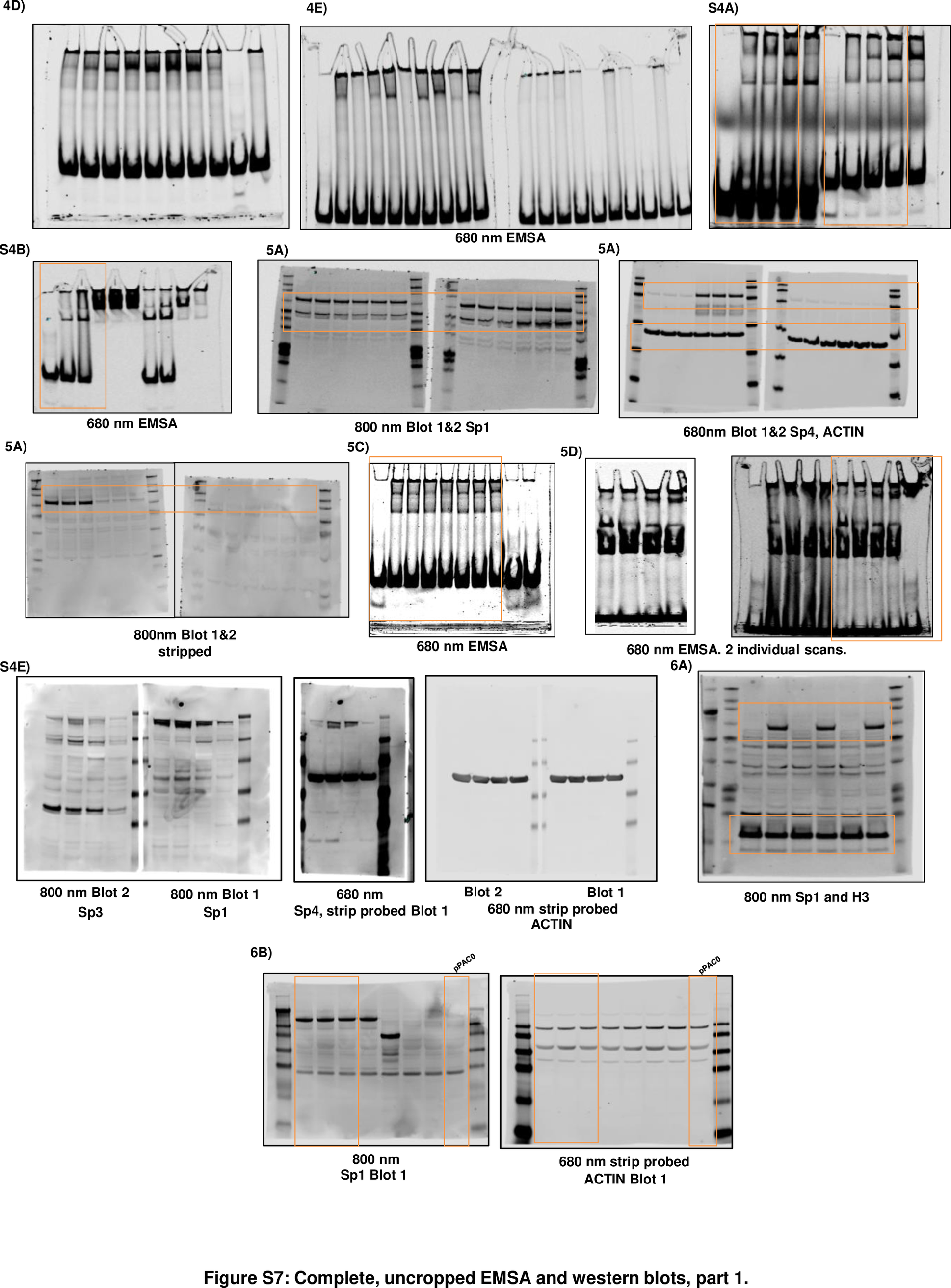

Supplement: S7 Fig — Figure labels used for all uncropped blots here are same as the figure labels used in the cropped blots. 680 and 800 nm indicates the LICOR IR dye channel used for scanning. Indicated blot number can be used to trace stripping and reprobing order. (TIF) [file ppat.1012984.s007.tif]

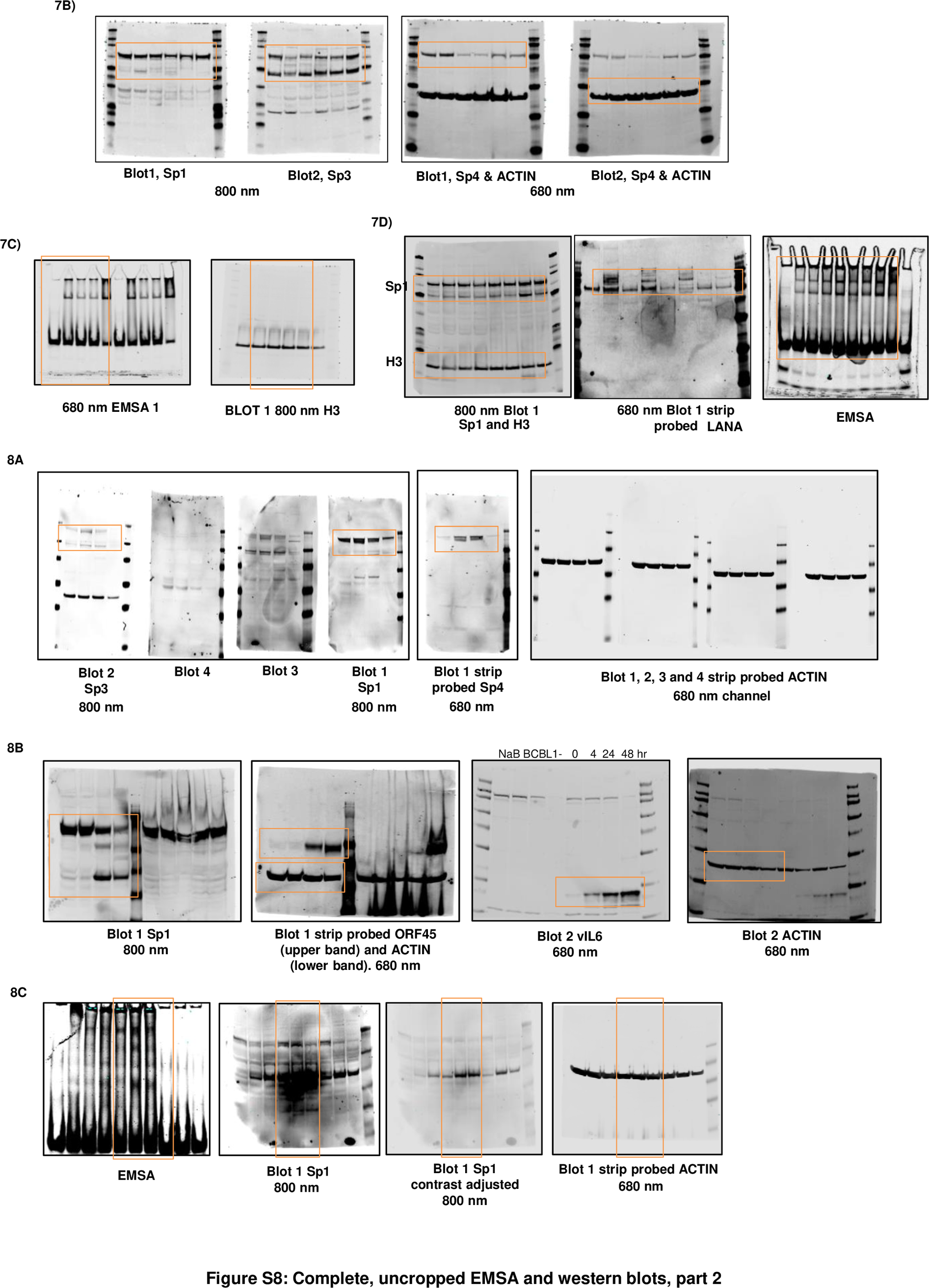

Supplement: S8 Fig — Figure labels used for all uncropped blots here are same as the figure labels used in the cropped blots. 680 and 800 nm indicates the LICOR IR dye channel used for scanning. Indicated blot number can be used to trace stripping and reprobing order. (TIF) [file ppat.1012984.s008.tif]

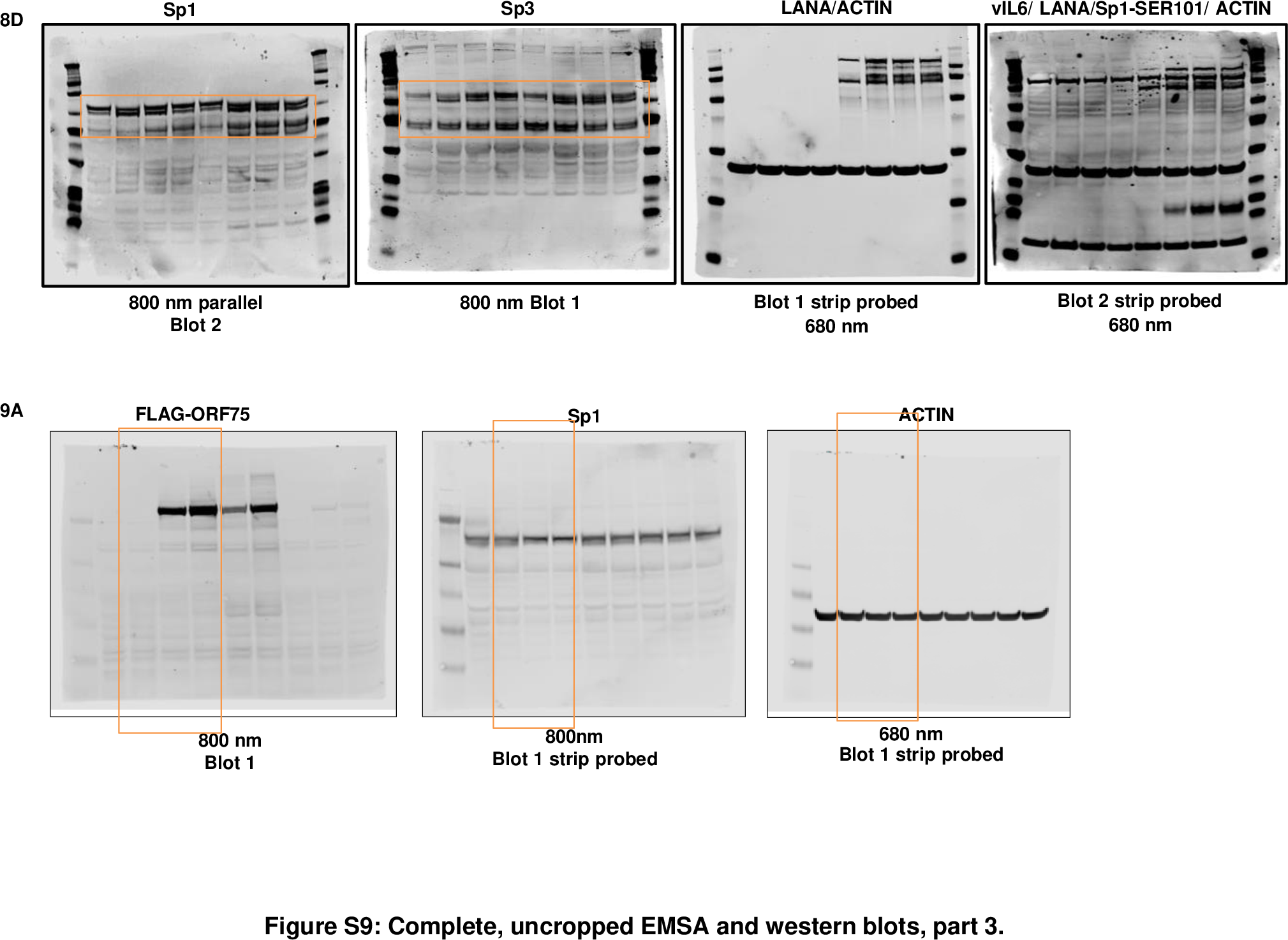

Supplement: S9 Fig — Figure labels used for all uncropped blots here are same as the figure labels used in the cropped blots. 680 and 800 nm indicates the LICOR IR dye channel used for scanning. Indicated blot number can be used to trace stripping and reprobing order. (TIF) [file ppat.1012984.s009.tif]
