# Supplementary material for: The elevated expression of ORF75, a KSHV lytic gene, in Kaposi sarcoma lesions is driven by a GC-rich DNA cis element in its promoter region
Source: PLoS Pathog. 2025 Mar 17;21(3):e1012984. doi: 10.1371/journal.ppat.1012984 (PMC11981178; doi:10.1371/journal.ppat.1012984)
Supplement: S1 Table — (DOCX) [file ppat.1012984.s010.docx]

**S1 Table: List of antibodies used in the study**

|  |  |
| --- | --- |
| Anti-ORF75 | HAMB custom Rabbit polyclonal, Genscript Inc. |
| Anti-LANA | HAMB custom mouse monoclonal. Advanced Biotechnologies Inc., Columbia, MD |
| Anti-Sp1 | Proteintech 21962-1-Ap, rabbit polyclonal. |
| Anti-Sp1 | Santa Cruz sc-420-1C6, mouse monoclonal |
| Anti-Sp1 | Santa Cruz sc-17825-E-3, mouse monoclonal |
| Anti-Sp3 | Proteintech 26584-1-Ap, rabbit polyclonal. |
| Anti-ORF45 | Abcam, 2D4A5, Mouse monoclonal. |
| Anti-vIL6 | HAMB custom mouse monoclonal |
| Anti-FLAG | Cell Signaling Technology, 14793 |
| Anti-Sp4 | Sc-390124 (B-1), Santa Cruz |
