# Supplementary material for: The elevated expression of ORF75, a KSHV lytic gene, in Kaposi sarcoma lesions is driven by a GC-rich DNA cis element in its promoter region
Source: PLoS Pathog. 2025 Mar 17;21(3):e1012984. doi: 10.1371/journal.ppat.1012984 (PMC11981178; doi:10.1371/journal.ppat.1012984)
Supplement: S2 Table — (DOCX) [file ppat.1012984.s011.docx]

| Gene  **S2 Table: List of primers used in the study** | Primer sequence | Reference | Experiment |
| --- | --- | --- | --- |
| RTA | F: **TTGCCAAGTTTGTACAACTGCT**  R: **ACCTTGCAAAGACCATTCAGAT** | Wang et al. 2020 [1] | qPCR |
| vIL6 | F: **CTGTTACCGTACCGGCATCT**  R: **GGGTGGACTGTAGTGCGTCT** | Wang et al. 2020 [1] | qPCR |
| Actin | F: **CCTTCCTGGGCATGGAGT**  R: CAGGGCAGTGATCTCCTTCT | Wang et al. 2020 [1] | qPCR |
| 18S | F: GCCCGAAGCGTTTACTTTGA  R: TCCATTATTCCTAGCTGCGGTATC | Veeranna et al. 2012 [2] | qPCR |
| ORF72 | F: CATTGCCCGCCTCTATTATCA  R: ATGACGTTGGCAGGAACCA | Vladimirova et al. 2023 [2] | qPCR |
| LANA | F: GTGACCTTGGCGATGACCTA  R: CAGGAGATGGAGAATGAGTA | Veeranna et al. 2012 [2] | qPCR |
| ORF74 | F: CAAGCAGGCCATGTGTTATG  R: AGCACCACAGCAACAATCAC | This study | qPCR |
| PAN | F: CGGTGTTTTGGCTGGGTTT  R: AAACCTTGCCGTCTGGTCACT | Vladimirova et al. 2023 [3] | qPCR |
| ORF75 coding | F: TATACAAGCCGTACGGGCAC  R: CTCGAAGTGGGAGGTCTTCG | Vladimirova et al. 2023 [3] | qPCR |
| *ORF56* | F: GGTCCACAGATTCCCGTCAA  R: GGGGAGTGATGGAGCAGTTC | This study | qPCR |
| *ORF45* | F: CGTCCGGAGAGTTGGAACTG  R: GCGATCGTCGACCTGACAT | This study | qPCR |
| *ORF39* | F: TTTCCACCGAGTCAGCAGTG  R: ACACGTACTTATGGCAGCCT | This study | qPCR |
| *ORF30-33* | F: GCATTTCCTGCCCTGGTTCT  R: CAGCCTCTGCAGTTCCTCC | This study | qPCR |
| *ORF75 endogenous* | F: ATCACTCTCCAACCACAGCC  R: CACAGGTTGCTCTGCAGAGT | This study | qPCR |
| *K15_EXON1* | F: GGCCCTACTGGTATGTTTTTGG  R: CCAGGATGAAGGCCATTTAGG | This study | qPCR |
| *K15_EXON3* | F: GTCCACATGTCAAACACCTGG  R: ATGGCAAGCTGAGAGTGAAGC | This study | qPCR |
| *ORF57* | F: ACGAATCGAGGGACGACG  R: CGGGTTCGGACAATTGCT | This Study | qPCR |
| *DHFR* | F: TCGCCTGCACAAATAGGGAC  R: AGAACGCGCGGTCAAGTTT | Kaeser et al. 2002 [4] | ChIP-qPCR |
| *P1-ORF75* | F: ACTCCTGCAGTCCTAGCTCT  R: CACCGCTCCTTGGATATCCC | This study | ChIP-qPCR |
| *P2-ORF75* | F: GGGATATCCAAGGAGCGGTG  R: GCACACTCACTCTCGTACCC | This study | ChIP-qPCR |
| *P3-ORF75* | F: ATCACTCTCCAACCACAGCC  R: ACTCTGCAGAGCAACCTGTG | This study | ChIP-qPCR |
| *P4-ORF75* | F: CACAGGTTGCTCTGCAGAGT  R: CCTCCACGACCACAGACTTT | This study | ChIP-qPCR |
| *P5-ORF75* | F: AAAGTCTGTGGTCGTGGAGG  R: TCACCACAGCCAGACCAATC | This study | ChIP-qPCR |
| *P6-ORF75* | F: TCTGGCTGTGGTGATGGTTC  R: GGGAGCCAACTACTGAACGA | This study | ChIP-qPCR |

**References:**

1. Wang V, Davis DA, Deleage C, Brands C, Choi HS, Haque M, Yarchoan R. Induction of Kaposi's Sarcoma-Associated Herpesvirus-Encoded Thymidine Kinase (ORF21) by X-Box Binding Protein 1. Journal of virology. 2020;94(5).

2. Veeranna RP, Haque M, Davis DA, Yang M, Yarchoan R. Kaposi's sarcoma-associated herpesvirus latency-associated nuclear antigen induction by hypoxia and hypoxia-inducible factors. Journal of virology. 2012;86(2):1097-108.

3. Vladimirova O, Soldan S, Su C, Kossenkov A, Ngalamika O, Tso FY, et al. Elevated iNOS and 3'-nitrotyrosine in Kaposi's Sarcoma tumors and mouse model. Tumour Virus Res. 2023;15:200259.

4. Kaeser MD, Iggo RD. Chromatin immunoprecipitation analysis fails to support the latency model for regulation of p53 DNA binding activity in vivo. Proceedings of the National Academy of Sciences of the United States of America. 2002;99(1):95-100.
