# Supplementary material for: The elevated expression of ORF75, a KSHV lytic gene, in Kaposi sarcoma lesions is driven by a GC-rich DNA cis element in its promoter region
Source: PLoS Pathog. 2025 Mar 17;21(3):e1012984. doi: 10.1371/journal.ppat.1012984 (PMC11981178; doi:10.1371/journal.ppat.1012984)
Supplement: Attachment — Submitted filename: Response to Reviewers250116ry_250120AN_Final.docx [file ppat.1012984.s015.docx]

We thank the Editor and the Reviewers for the careful review of our manuscript entitled “The Elevated Expression of ORF75, a KSHV Lytic Gene, in Kaposi Sarcoma Lesions is Driven by a GC-rich DNA *cis* Element in its Promoter Region”. We are pleased that it is found to have merit and is in general carefully performed. We have now revised the manuscript to address the specific comments. Please note that in the response to a comment of Reviewer 3, we have modified the title somewhat and placed the word “Lytic” after KSHV. Below is a point-by-point description of the changes we have made in response to these comments.

**Reviewer #1:**

**Nair et al. show that KHSV ORF75, an annotated lytic protein, is pervasively expressed in latently infected KS skin lesions. They examine the distinct behavior of ORF75’s promoter in several different cell lines, demonstrating that its activity is most prominent in endothelial and less so in B cells. This may be due to differences in expression or activity of the SP1 transcription factor between cell types, and their promotor deletion mapping experiments identify key SP1 binding sites within the ORF75 promoter and suggest the presence of a repressive element that is active in B cells. The combined promoter deletion mapping experiments in mammalian cells and SP1 complementation assays in SP1-deficient drosophila cells nicely support an SP1-dependent mechanism. They also pursue SP1 gel shifts and overexpression experiments in mammalian cells (these are somewhat hard to interpret given that it’s not clear that SP1 is limiting—it could be more informative to deplete SP1 in these cells and show loss of ORF75 promoter activity). Finally, they show that ORF75 overexpression activates several viral promoters, including RTA and ORF75 itself. These data suggest new roles in viral latency and gene regulation for this annotated tegument protein and raise interesting new models for both latency and reactivation in KSHV.

Despite the interesting conceptual advance, there are concerns about how the data are presented (e.g., ‘breaking’ of Y-axes rather than converting to log scale) or the lack of error bars. In some cases, the legends suggest technical rather than biological replicates, though it is not easy to tell. Please confirm that all experiments (main and supplemental) have at least 3 biological replicates; this should be stated for blots/gel shifts and represented as error bars in graphs.**

**Author’s Responses to Reviewer #1 Comments**

**General Comments**
We thank Reviewer 1 for the thoughtful review. We believe that the strongest evidence linking Sp1 to ORF75 promoter activity is the experiment in Sp protein-deficient SL2 cells. However, as noted, our other experiments were conducted in cells where Sp1 was not limiting, complicating interpretation. To address this, we added new ORF75 promoter experiments using Sp1-knockdown 293T cells **(Fig. 6D & E)**. These results show that Sp1 depletion significantly reduces ORF75 basal promoter activity, correlating with Sp1 knockdown levels. This reinforces the evidence for Sp1's role in ORF75 regulation. We also confirm that all blots and gel shift assays were performed with at least three biological replicates, as now stated in the revised manuscript.

1. **Data Presentation Issues:**

a. **In Figure 2b, the legend indicates 3 time points were taken at day 30, 35, and 40, but the graph does not show multiple timepoints. What timepoint is represented by the 3 data points in each bar of that graph? Also, the graph is presented with a broken y-axis, which appears to have distorted the data above the break as well as the error bar spanning the break for ORF75 in TIME.219 cells. The data should be presented on a log-scale to allow these comparisons without distortion.**

**Author’s Response:** The time points shown in the original **Fig 2B** indicate the time that the established TIME.219 line was samples for study, However, the line is considered established stable during this period, and to avoid confusion, these have been replaced in the revised manuscript with a simple mean and SD error bars. We apologize for the error in the broken y-axis and have corrected this by switching to standard bar graphs and excluding PAN gene expression for clarity.

b. **Figure 2c is missing error bars or lacks 3 biological replicates.**

**Author’s Response:** We have added trendlines for individual biological replicates for each time point to address this concern.

c. **Figure S3, panels c-f are either missing error bars or lack 3 biological replicates.**

**Author’s Response:** These experiments were performed with two replicates, as noted in the figure legend. These experiments were performed to examine potential contributions of NRF2, HIF1, and HIF2 to ORF75 basal expression using multiple approaches (e.g., NRF2 inducers H_2_O_2_, tBHQ and HIF1/2 inducers like hypoxia). While not done in triplicate, these were essentially negative results, and we believe the experiments done are sufficient to discount the roles of these factors. Moreover, we provide additional evidence for the lack of contribution for these factors by mutating consensus sites for NRF2 and HIFs **(Fig. 3E)**. Finally, the subsequent data showing that Sp1 is the major factor inducing ORF75 expression provides further evidence that these other factors are not the main drivers of ORF75 repression.

d. **In Figure 4a and Figure 5a, for example, the fold changes given are used inconsistently, with those in Figure 5a matching the y-axis value but Figure 4a does not. Please correct or clarify.**

**Author’s Response:** We have standardized the axes of all promoter luciferase assay graphs to relative luciferase units. Fold changes relative to control are indicated atop each bar for clarity, as noted in the figure legend.

e. **In Figure 4f, no error bars are shown, and the broken y-axis appears to be distorting the data.**

**Author’s Response:** This figure has been moved to supplementary materials (now S4c), the y-axis standardized, and two experimental replicates presented separately.

f. **Figure 8h, data appears distorted above the broken y-axis.**

**Author’s Response:** This is now Figure 9h. In this revised figure, the broken y-axis has been replaced with a standard y-axis to eliminate distortion. Also, all values are compared with non-transfected 293T-BAC16 cells.

**Reviewer #1: Minor Comments**

**The manuscript could be streamlined to remove some of the data that are tangential to the major claims. E.g., data in Fig. S5 and Fig.7 that aren’t quite developed enough to draw clear conclusions.**

**Author’s Response:** We appreciate the suggestion. In response, we have streamlined the section describing **Fig 7** somewhat (now Fig 8) and taken out redundant material. Also, we have removed data about OCT2, which we agree is unlikely to play a role in the repression in B cells.

1. **The different binding intensity in the gel shift in Fig. 6C could be accounted for by modest expression differences of Sp1. I would temper the quantitative claims here.**

**Author’s Response:** The text has been modified accordingly.

2. **It would be clearer to show Figure 6e together with the corresponding data from HEK293T cells currently in Figure 4a.**

**Author’s Response:** While we recognize the connection between these data sets, presenting them together would add complexity. Instead, we have maintained a stepwise presentation: epithelial cells **(Fig. 4A)**, SL2 cells **(Fig. 6A)**, endothelial and B-cells **(Fig. 7A)**, and comparative analysis of KSHV-relevant cell lines **(Fig. 7E)**.

3. **In Figure 8, a supernatant transfer or other measurement of productive reactivation would be useful to know whether ORF75-induced activation of the RTA promoter is sufficient for reactivation into a productive lytic cycle.**

**Author’s Response:** Thank you for this suggestion. We haven’t performed a supernatant transfer assay to test for production of infective virions for this experiment. We are actively pursuing the function of ORF75 during latency, latency to lytic transitioning and lytic cycle. We hypothesize that ORF75 expression primes the latent viral genome for transitioning to lytic cycle. However, other triggers are required to reactivate lytic replication followed by production of infective progeny.

4. **The authors may consider examining or discussing the expression of K15, which shares an annotated polyA site with ORF75.**

**Author’s Response:** We have now analyzed K15 expression in infected endothelial, epithelial, and B-cell lines. Our qPCR results indicate that ORF75 is expressed at much higher levels than K15 in TIME.219 cells, highlighting the distinct promoter activity of ORF75. These data are presented in **Fig. S2B**.

5. **It could be interesting to speculate in the discussion on potential parallels between their observations of ORF75 and the behavior of EBV ZTA, given their data suggests ORF75 and ORF50 are mutually activating.**

**Author’s Response:** Thank you for this suggestion. We have briefly discussed potential parallels between KSHV ORF75 and EBV ZTA in the revised discussion, focusing on the synergy between ORF75 and RTA, akin to the interaction between BNRF1 and ZTA.

**2) The authors are commended for exploring their phenotypes in multiple cell lines; however, the frequent cell line switching makes direct comparisons hard, particularly in Fig.6: Fig. 6A makes arguments about ORF75 promoter activity being increased in TIVE, then 6B compares Sp1 expression between B and endothelial cells (but uses HUVEC, not TIVE), then 6C uses TIME as the endothelial cell representative for gel shifts. Need one endothelial line carried through for direct comparisons.**

**Author’s Response:** We agree that consistent use of an endothelial cell line improves clarity. We have added new figures **(Fig. 7B and C)** showing Sp1 levels in B-cells, endothelial cell lines (TIME, TIVE), and epithelial cells (293T). Additionally, we performed EMSA for the proximal Sp1 element with these cell lines for direct comparison.

**Reviewer #2:** **This is an interesting study on the regulation of KSHV ORF75 gene expression by Nair et al. The ORF75 gene was found to be a lytic gene expressed upon reactivation of KSHV from infected B lymphoma cells. The authors here demonstrated that ORF75 can also be expressed in latently infected cells in KS tumors, and in infected endothelial and epithelial cells. The expression is mediated by Sp1 element binding proteins located upstream of the ORF75 promoters. Interestingly, the authors demonstrated that its expression was suppressed by repressors binding to the ORF75 promoter, which is quite interesting. However, this potential repressive factor still remained uncharacterized. The ORF75 protein was also found to upregulate the expression of several KSHV genes, including activation of its own promoter and RTA ORF50 promoter. The experiments in the paper were very well executed, the molecular analyses of the promoter elements and their binding proteins were detailed, and meticulously and logically carried out.** **The results provided are convincing. However, there are a number of points that the authors need to address. One is the fact that ORF50 was usually not expressed in latently infected endothelial cells, and now the authors demonstrated that ORF75 can effectively activate ORF50 promoter. If this is in fact true, then why did these infected cells remained latent and did not go through lytic reactivation since ORF50 expression is the key to lytic reactivation. Another is the variability of ORF75 expression in different endothelial, epithelial and B cell lines. For example, TIME cells are telomerase immortalized, what are effects of the process in immortalization of these cells on ORF75 promoter? Since the expression of various cellular factors are variable among different cell lines as demonstrated by some of the results shown, what causing this variability should be addressed.**

**Author’s Responses to Reviewer #2 Comments**

**General Comments**

We thank Reviewer 2 for the complementary assessment and the thoughtful comments. These comments were amplified in the specific points below, and we have addressed them there.

**1. There were very little RTA expression in the latently infected TIME.219 cells as shown in Fig. 1B, since the authors showed activation of RTA promoter in Fig. 8, why then RTA expression was not elevated in results shown in Fig. 1. In addition, since Fig. 8 showed that ORF75 also activated ORF57 promoter, the expression of ORF57 should also be analyzed in Fig. 1b in TIME.219 cells.**

**Author’s Response:** We have shown that overexpressing ORF75 in latently infected cells or cells containing the RTA promoter causes activation of RTA and other promoters. However, this does not indicate how much ORF75 RNA or protein is required for RTA promoter induction. It is also possible that ORF75 expression was insufficient to significantly induce RTA activation in TIME.219 cells. The Sp1 protein regulates the expression of the **ORF75** gene, and its activity is modulated throughout the cell cycle, responding to various signals such as starvation, shear stress and redox state. Variations in Sp1 transcriptional activity driven by these signals can affect ORF75 expression. While the TIME.219 cell line maintained under standard growth conditions is suited to assess the basal expression of the ORF75 gene, it does not measure the inducibility of the promoter. In theTIME.219 cell line, we detected 2–3-fold higher RTA RNA relative to LANA RNA, indicating some elevation in RTA expression, though not substantial. Finally, ORF57 RNA expression data has now been included in **Fig. S2B**, demonstrating significant induction of ORF57 in TIME.219 cells compared to BCBL-1 cells.

**2. ORF57 expression in epithelial iSLK-BAC16 cells was not robust as compared to TIME.219, is it possible that there are additional suppression factors in iSLK-BAC16 cells that are similar to those found in B cells. In other words, there could be cell lines specific transcriptional or suppressor factors that down or upregulate ORF75 promoter expression.**

**Author’s Response:** In our paper, we have tried to explore in depth the difference in ORF75 expression between B cells and endothelial cells. (We believe the reviewer is referring to ORF75 rather than ORF57). However, as the Reviewer points out, it is likely that there are a variety of elements, including repressive elements, affecting its expression in other lines. The variable expression of ORF75 across cell lines likely reflects a variety of differences in cellular environments, which influence basal latent expression. Our findings indicate that Sp proteins interact with proximal Sp1 elements activating promoter in all cell lines. However, robust ORF75 expression also relies on distal regulatory regions, including CAAT boxes and Sp1-like sites. These Sp1 elements in the ORF75 promoter interact differently in various cell lines, potentially due to repressors or cofactors limiting Sp1 access. For instance, alternate Sp1 forms in B cells might serve as repressors. Although identifying these factors is beyond the current study, we have highlighted their likely contributions.

**3. Studies of ORF75 expression were mostly carried out in infected immortalized cells, it should also be carried out with primary endothelial cells infected cells such as HUVEC.**

**Author’s Response:** We have now included data on ORF75 expression in *de novo* infected primary Human Dermal Microvascular Endothelial Cells (HDMVEC) **(Fig. S2C)**. ORF75 expression in HDMVEC mirrors trends observed in *de novo* infected TIME cells.

**4****. Fig. 4A, what is the reason why mutations of the ARE sites with p75-&4_mARE 200 led to higher expression activities as compared to the wt p-75 200bp promoter construct?**

**Author’s Response:** Mutating the antioxidant response elements (AREs) in the ORF75 promoter led to a small but significant increase in promoter activity. We do not know the reason for this but can speculate. The proximal ARE elements may function as repressive sites. The regulatory role of AREs depends on their binding partners; for example, NRF2 typically activates AREs but can mediate repression when dimerized with sMAF proteins. It is an interesting observation and worth further exploration.

Reference: Raghunath A, Sundarraj K, Nagarajan R, Arfuso F, Bian J, Kumar AP, Sethi G, Perumal E. Antioxidant response elements: Discovery, classes, regulation and potential applications. Redox Biol. 2018 Jul;17:297-314. doi: 10.1016/j.redox.2018.05.002. Epub 2018 May 7. PMID: 29775961.

**5. Fig. 4D. Gel shift analyses were carried out with cells overexpressing various Sp factors, results without overexpression should also be shown. Was Sp1 also over expressed in SpS4?**

**Author’s Response:** Gel shift assays using 293T lysates without Sp factor overexpression are now shown in **Fig S4A** and **Fig S4B**. Additionally, a supershift assay for Sp1 is included in **Fig 5D**. We have now included data on overexpression levels of Sp proteins. We did not observe an increase in Sp1 protein upon Sp4 overexpression as is observed in experiment **Fig 5A**.

**6. Fig. 4F. What is the explanation that the CHIP assay showed an enrichment of binding by both Sp1 and Sp2 to the P1 and P2 fragments, which are located in the ORF75 ORF and not in the promoter region?**

**Author’s Response:** Sp1 and Sp3 proteins are known to bind a variety of GC boxes. In this case, the consensus GC box critical for expression lies at -11 from the ATG. However, there are many Sp1-like regions with partial consensus in the promoter as well as in the coding region of ORF75 (for example one functional Sp1-like site based on promoter assays in SL2 cells is located +85 of ATG). This aligns with a “ramp model” of activation, where Sp1 binds sequential elements, including Sp1-like sites within the coding sequence, to form a super enhancer. The figure has been moved to supplementary materials **Fig S4C**.

**Reference:** Y. Hasegawa, K. Struhl, Different SP1 binding dynamics at individual genomic loci in human cells, *Proc. Natl. Acad. Sci. U.S.A.*118 (46) e2113579118,

<https://doi.org/10.1073/pnas.2113579118> (2021).

**7. Fig. 5A. Why did overexpression of Sp3 enhance the expression of p75-T2 more than what was observed with overexpression of Sp1? Similarly, why did the overexpression of Sp4 enhance the expression of p75 better than Sp1 in HepG2 cells? This further supports the notion that different cellular factors are involved in enhancement and suppression of p75, which are highly cell type dependent and may even involve different mechanisms.**

**Author’s Response:** Sp1, Sp3, and Sp4 are transcription factors that can act as activators or repressors. The experiment in **Fig 5A** investigated whether Sp3 and Sp4 repress Sp1-driven promoter activity. We have now included data to show the overexpression protein levels (lower W.B panel **Fig 5A**). Overexpression of Sp3 and Sp4 increased promoter activity, indicating they cooperate with Sp1 elements in the ORF75 promoter to enhance transcription. Similarly, Sp1 overexpression further upregulated promoter activity, suggesting these proteins form complexes that hyperactivate the promoter depending on transcription factor availability.

While Sp1/Sp3 synergistically bind GC-box-rich promoters, Sp4 preferentially binds GT-boxes and may activate a specific site in the ORF75 promoter when Sp1/Sp3 levels are low, as suggested by the shift in promoter activity observed in **Fig 5A**. The p75-T2 promoter, being shorter than the full-length p75 promoter, may include specific Sp3 binding sites. However, dissecting Sp3's role is beyond this study. We agree with the reviewer that repression involves different factors, but promoter enhancement is driven by an Sp1-based complex involving Sp3 and Sp4 depending on cell type. To confirm Sp1's role, we show that Sp1 knockdown in 293T cells reduces ORF75 promoter activity, demonstrating that its basal expression closely correlates with Sp1 levels **Fig 6D & E**.

Reference: Bigger, Catherine B., Gardner, Paul, D, Sp1 and Sp3 Regulate Expression of the Neuronal Nicotinic Acetylcholine Receptor β4 Subunit Gene *Journal of Biological Chemistry, Volume 272, Issue 41, 25976 – 25982.

**8. Fig. 7A. Why was the 45 kDa Sp1 band not detected in BJAB cells? What is the possibility that KSHV induces the expression of 45 kDa Sp1 expression is some cell lines?**

**Author’s Response:** The 45-kDa Sp1 form accumulates in B cells during apoptosis and is essential for cell death. While uninfected healthy BJAB cells lack this form, in data not shown here, we have observed its accumulation in BJAB cells upon triggering apoptosis. As noted in the paper, we do see it in KSHV-infected B cells (e.g.PEL lines), likely due to higher caspase activity. This suggests KSHV infection drives 45-kDa Sp1 expression in these contexts.

**9. Fig. 8C. It is not clear why there was no LANA detected in 293-BAC16 cells.**

**Author’s Response:** We believe the reviewer referred to **Fig 6C** (now **Fig 7D**), which includes LANA western blot data for various cell lines. While LANA is detectable in 293T-BAC16 cells, its expression is weaker compared to other cell lines. A replicate blot with adjusted contrast has been added to the supplementary material for clarity.

**Other Issues**

**1. It is not clear what cell types were used to identify the transcriptional repressor of ORF57 in Fig S5A.**

**Author’s Response:** We believe the reviewer referred to ORF75 instead of ORF57. Repressor assays in **Fig S5A** were performed in 293T cells, where B-cell repressor factors are minimally expressed. This is now explicitly stated in the figure legend.

**2. Line 873 Fig.5 legend. It is not clear what the authors meant by “.....treatment during PRF75 promoter (p75-T2) luciferase assay in 293T-cell”. Does it mean that the treatment was added during the luciferase assay? It needs to be rephrased.**

**Author’s Response:** We have revised the phrasing: “ORF75 promoter (p75-T2) luciferase activity in the presence of Sp1-specific inhibitor Mithramycin A.”

**Reviewer #3:** **The manuscript by Nair et al. entitled “The Elevated Expression of ORF75, a Lytic KSHV Gene, in Kaposi Sarcoma Lesions is Driven by a GC-rich DNA cis Element in its Promoter Region” explores the elevated expression of KSHV ORF75, a lytic gene, in Kaposi Sarcoma (KS) lesions, where the virus usually maintains latency. Through RNAscope, the authors found that ORF75 RNA is highly expressed in cells positive for latency-associated nuclear antigen (LANA), indicating its prominent role even in latent phases. By employing luciferase reporter assays, they demonstrated that the transcriptional activation of ORF75 is driven by a GC-rich Sp1 cis-element in its promoter, along with distal CCAAT boxes. Sp proteins are shown to interact with these elements, activating the ORF75 promoter in endothelial cells, while a repressive factor in B cells limited its expression in these cells. The study also observed autoregulation, where ORF75 upregulates its expression and other KSHV genes, suggesting a feedback loop during viral replication. Additionally, the accumulation of forms Sp1 during viral latency and lytic phases in PEL cells and endothelial cells hints at a complex regulatory mechanism.
In summary, this work highlights the significant constitutive expression of ORF75 in KS lesions, driven by transcriptional mechanisms involving Sp1 proteins, which could account for its high levels in infected endothelial cells. The data presented in this manuscript convincingly support the hypothesis that ORF75 is highly expressed in Kaposi Sarcoma (KS) lesions due to the activity of a GC-rich Sp1 cis-element in its promoter. The findings regarding Sp1 protein interactions and their role in transcriptional regulation are robust. However, the specificity of Sp protein binding to the promoter elements could be further validated to strengthen the conclusions.**

**Author’s Responses to Reviewer #3 Comments**

**General Comments**

Response: We thank Reviewer 3 for his assessment that the manuscript addresses the high constitutive expression of ORF75 in KS and that the general findings of Sp1 protein and their role in transcriptional activation are robust, our response to concerns raised are below.

**Reviewer #3:**

1. **The electrophoretic mobility shift assay shows the retardation of the band due to the specific binding of probes to a protein. Since Sp1 binding site was included in the probes, it can be concluded that the retardation of band mobility is due to Sp1 binding. Additionally, using cold competitors to abolish binding suggests that the band is specific to the probe. However, this experiment will benefit from demonstrating the specificity of Sp1 binding to the DNA by utilizing anti-Sp1 antibodies to shift the Sp1-Probe complex further. Although the Sp1 mutated probe suggests that the shift in the band mobility is due to the Sp1 binding, having that additional lane will strengthen the data.**

**Author’s Response:** Thank you for the suggestion. We have added an additional blot showing a supershift with several Sp1 antibodies, which presumably bind to different domain of Sp1 protein. **(Fig. 5D).**

2. **Figure 5B: The claim of more Sp1 than Sp3 and Sp4 in the nucleus of HUVEC cells seems inaccurate as BCBl-1 cells also show high levels of Sp1 ad compared to Sp3 and Sp4. Fractionation of cytoplasmic and nuclear fractions to demonstrate the proportion of these Sp proteins in these two cell lines, along with the control of accurate fractionation, might provide a better estimation of a proportionate localization.**

**Author’s Response:** We agree with the reviewer’s suggestion that this is an overinterpretation. This point is not essential for the manuscript, and we have now removed the previous **Fig 5B** and have rephrased our sentence.

3. **The levels of Sp1 protein through the immune blot in these MA-treated cells used for luciferase assay will show that, indeed, increasing concentrations of MA determine the levels of Sp1.**

**Author’s Response:** Thank you for your suggestion. MA is a well-studied Sp protein inhibitor. While previous studies have demonstrated that MA specifically degrades Sp1 and reduces its binding to the GC box, we unfortunately lost the protein samples for this experiment and could not verify Sp1 levels. However, we have added a Sp1 knockdown experiment in the manuscript in **Fig 6D and 6E**, which shows a similar effect to MA treatment and may even be more specific.

4. **Figure 5D: The use of anti-Sp1 and Sp3 antibodies to show their binding specificity does not convincingly show that the indicated Sp1 band is specific to Sp1 binding. Instead, the increasing amounts of anti-Sp1 antibodies in the mix have reduced the band intensity, suggesting that anti-Sp1 antibodies might be competing with the probe binding. Utilizing purified Sp1 and Sp3 proteins for these assays will be more convincing.**

**Author’s Response:** We have now used multiple Sp1 and Sp3 antibodies and performed supershift assays on the P75 50-nt DNA probe containing the pSp1 element. This is shown in a new figure, **Fig 5D**. As previously observed, using Sp1 and Sp3 antibodies (AP, Proteintech) led to a disappearance of binding without a shift for anti-Sp1, but not for anti-Sp3. With other Sp1 antibodies, we now observe a supershift with the ORF75 proximal Sp1 element probe. Additionally, we have included a Sp1 knockdown experiment in 293T cells **(Fig 6D & E)**, showing a significant reduction in ORF75 promoter activity, which correlates with the level of Sp1 expression.

**Minor comments:**

We have incorporated all other minor suggestions from Reviewer #3 into the manuscript. We thank the reviewer for their thorough review and valuable suggestions, which have improved the conclusions and readability of the manuscript.

1. **Figure 3C**: The luciferase units seem low unless multiplication factors exist.
   **Author’s Response**: Thank you for your comment. The figure has been updated with the correct histogram.
2. **Figure S3C**: These fold changes are not highly significant, mainly when no error bars exist. Error bars should be shown on other panels, as well as other supplemental figures.
   **Author’s Response**: As described in the response to Reviewer 1 (comment c), Figure S3 presents negative results showing that other factors do not affect ORF75 promoter activation. Figure S3C utilized overexpression of the factors, and even under these conditions, we see little to no change in ORF75 promoter activity. Given that the result is essentially negative, we are unsure whether repeated experiments are necessary here. Since small but insubstantial changes were observed, we have updated the text to state that there was “little or no enhancement of activity.”
3. **In the title**, the term “cis” should be italicized per convention.
   **Author’s Response**: cis has been italicized in the manuscript title.
4. **Perhaps “lytic”** should come after KSHV in the title.
   **Author’s Response**: The term "lytic gene" was moved after "KSHV" in the title.
5. **Line 28**: The term “production” should be “expression” because genes are expressed rather than produced.
   **Author’s Response**: The term "production" has been replaced with "expression" in Line 28 of the updated manuscript text.
6. **Line 38**: Bold parenthesized references.
   **Author’s Response**: References have been bolded in Line 38 of the updated manuscript text.
7. **Line 51**: CRISPR and siRNA techniques are mentioned, but only about knocking out ORF75. Since siRNA is mentioned, perhaps knocking down should be included.
   **Author’s Response**: The text has been updated to: “Knocking out or suppressing ORF75 expression in infected iSLK cells through CRISPR or siRNA techniques was shown to lead to either complete abolition or significant reduction in viral genome replication, respectively.”
8. **Line 81**: The word “gene” is missing after “lytic.”
   **Author’s Response**: The text was updated to include "lytic gene" in Line 81 of the updated manuscript text.
9. **Line 83**: Add a space between the period and “To…”
   **Author’s Response**: A space was added after the period in Line 83 of the updated manuscript text.
10. **Line 85**: Remove the space after “1A” within the parentheses.
    **Author’s Response**: The space after "1A" was removed in Line 85 of the updated manuscript text.
11. **Line 154**: “…within it” is vague and would be improved by explicitly stating “…within the promoter region.”
    **Author’s Response**: The text has been updated to read “within the promoter region” instead of “within it” in Line 162 of the updated manuscript text.
12. **Lines 306, 310, 316, 324**: The abbreviation for the cell line used should be SL2, not Sl2.
    **Author’s Response**: The abbreviation "Sl2" was corrected to "SL2" in Lines 267, 272, 277, and 286 of the updated manuscript text.
13. **Line 353**: “SP1” should be “Sp1” (lowercase “p”).
    **Author’s Response**: The text was updated to “a promoter area distal to the pSp1 element,” correcting "SP1" to "pSp1" (proximal Sp1) in Line 358 of the updated manuscript text.
14. **Line 421**: “lessor” should be “lesser.”
    **Author’s Response**: "Lessor" was replaced with "lesser," updating the sentence to “lower induction of vIL6 RNA” in Line 420 of the updated manuscript text.
15. **Line 426**: “access” should be “assess.”
    **Author’s Response**: "Access" was corrected to "assess" in Line 425 of the updated manuscript text.
16. **Line 455**: Insert “a” between “that” and “different.”
    **Author’s Response**: "A" was inserted between “that” and “different” in Line 452 of the updated manuscript text.
17. **Line 536**: “It’s…” is vague and would be improved by explicitly stating, “The lower constitutive expression of the ORF75 promoter…”
    **Author’s Response**: The text was updated to state, “The lower constitutive expression of ORF75 promoter in B cells” in Line 532 of the updated manuscript text.
18. **Line 592**: “Bacillus subtilis” should be italicized per convention.
    **Author’s Response**: Bacillus subtilis was italicized in Line 604 of the updated manuscript text.
19. **Line 659**: Remove the “n” in the word “ration.”
    **Author’s Response**: The letter "n" was removed, correcting the word to "ration" in Line 669 of the updated manuscript text.
20. **Line 724**: A reference is made to “(Hannah & Davis),” but no year is given, and the citation appears to be missing from the References section.
    **Author’s Response**: The proper reference, including the year, was added in Line 734 of the updated manuscript text.
21. **Figure 1C**: The green dashed-line box appears smaller than the area in the zoomed-in image.
    **Author’s Response**: The green dashed box was corrected to match the size of the zoomed-in image in Figure 1C.
22. **Figure 2B**: The key indicates that BCBL-1 is represented in blue but is actually in purple.
    **Author’s Response**: The color key was corrected in Figure 2B.
23. **Figure 6C**: An asterisk is adjacent to the LANA blots in the WB panel. What is this asterisk supposed to (if anything) indicate?
    **Author’s Response**: The asterisk indicates a non-specific band present in uninfected cells when probed with the LANA antibody. This has been clarified in the updated legend for Figure 7D of the updated manuscript figures.
24. **Figure S3B**: The x-axis appears to have markers for labels between each cell line dataset. Is this an error or are there missing labels (and data)?
    **Author’s Response**: The figure has been updated in the new MS figure. The labels in the x-axis showed pGL3 vector control expression for each cell line. As these are different cell lines, the y-axis showed fold change over pGL3 for each line instead of RLU units. The label markers correspond to pGL3 that was set to 1.
25. **Figure S3C**: The “3” is missing in the y-axis label for “pGL3.”
    **Author’s Response**: The figure was updated accordingly.
26. **Figure S4B**: This panel does not appear explicitly mentioned in the article's main text, but it would be worth stating that this western blot was performed to verify the expression of Sp proteins.
    **Author’s Response**: The blot was replaced in the figure, and its purpose was discussed in the Results section.
