# Supplementary material for: The elevated expression of ORF75, a KSHV lytic gene, in Kaposi sarcoma lesions is driven by a GC-rich DNA cis element in its promoter region
Source: PLoS Pathog. 2025 Mar 17;21(3):e1012984. doi: 10.1371/journal.ppat.1012984 (PMC11981178; doi:10.1371/journal.ppat.1012984)
Supplement: Attachment — Submitted filename: Nair et al_rev_10-24.docx [file ppat.1012984.s013.docx]

This is an interesting study on the regulation of KSHV ORF75 gene expression by Nair et al. The ORF75 gene was found to be a lytic gene expressed upon reactivation of KSHV from infected B lymphoma cells. The authors here demonstrated that ORF75 can also be expressed in latently infected cells in KS tumors, and in infected endothelial and epithelial cells. The expression is mediated by Sp1 element binding proteins located upstream of the ORF75 promoters. Interestingly, the authors demonstrated that its expression was suppressed by repressors binding to the ORF75 promoter, which is quite interesting. However, this potential repressive factor still remained uncharacterized. The ORF75 protein was also found to upregulate the expression of several KSHV genes, including activation of its own promoter and RTA ORF50 promoter. The experiments in the paper were very well executed, the molecular analyses of the promoter elements and their binding proteins were detailed, and meticulously and logically carried out. The results provided are convincing. However, there are a number of points that the authors need to address. One is the fact that ORF50 was usually not expressed in latently infected endothelial cells, and now the authors demonstrated that ORF75 can effectively activate ORF50 promoter. If this is in fact true, then why did these infected cells remained latent and did not go through lytic reactivation since ORF50 expression is the key to lytic reactivation. Another is the variability of ORF75 expression in different endothelial, epithelial and B cell lines. For example, TIME cells are telomerase immortalized, what are effects of the process in immortalization of these cells on ORF75 promoter? Since the expression of various cellular factors are variable among different cell lines as demonstrated by some of the results shown, what causing this variability should be addressed.

There are a number of points that the authors need to address:

1. There were very little RTA expression in the latently infected TIME.219 cells as shown in Fig. 1B, since the authors showed activation of RTA promoter in Fig. 8, why then RTA expression was not elevated in results shown in Fig. 1. In addition, since Fig. 8 showed that ORF75 also activated ORF57 promoter, the expression of ORF57 should also be analyzed in Fig. 1b in TIME.219 cells.
2. ORF57 expression in epithelial iSLK-BAC16 cells was not robust as compared to TIME.219, is it possible that there are additional suppression factors in iSLK-BAC16 cells that are similar to those found in B cells. In other words, there could be cell lines specific transcriptional or suppressor factors that down or upregulate ORF75 promoter expression.
3. Studies of ORF75 expression were mostly carried out in infected immortalized cells, it should also be carried out with primary endothelial cells infected cells such as HUVEC.
4. Fig. 4A, what is the reason why mutations of the ARE sites with p75-&4_mARE 200 led to higher expression activities as compared to the wt p-75 200bp promoter construct?
5. Fig. 4D. Gel shift analyses were carried out with cells overexpressing various Sp factors, results without overexpression should also be shown. Was Sp1 also over expressed in SpS4?
6. Fig. 4F. What is the explanation that the CHIP assay showed an enrichment of binding by both Sp1 and Sp2 to the P1 and P2 fragments, which are located in the ORF75 ORF and not in the promoter region?
7. Fig. 5A. Why did overexpression of Sp3 enhance the expression of p75-T2 more than what was observed with overexpression of Sp1? Similarly, why did the overexpression of Sp4 enhance the expression of p75 better than Sp1 in HepG2 cells? This further supports the notion that different cellular factors are involved in enhancement and suppression of p75, which are highly cell type dependent and may even involve different mechanisms.
8. Fig. 7A. Why was the 45 kDa Sp1 band not detected in BJAB cells? What is the possibility that KSHV induces the expression of 45 kDa Sp1 expression is some cell lines?
9. Fig. 8C. It is not clear why there was no LANA detected in 293-BAC16 cells.

Other issues:

1. It is not clear what cell types were used to identify the transcriptional repressor of ORF57 in Fig S5A.
2. Line 873 Fig.5 legend. It is not clear what the authors meant by “.....treatment during PRF75 promoter (p75-T2) luciferase assay in 293T-cell”. Does it mean that the treatment was added during the luciferase assay? It needs to be rephrased.
